# High-resolution behavioral time series of Japanese quail within their social environment

**DOI:** 10.1038/s41597-019-0299-8

**Published:** 2019-12-03

**Authors:** Jorge Martín Caliva, Rocio Soledad Alcala, Diego Alberto Guzmán, Raúl Héctor Marin, Jackelyn Melissa Kembro

**Affiliations:** 1 Universidad Nacional de Córdoba (UNC), Facultad de Ciencias Exactas, Físicas y Naturales, Instituto de Ciencia y Tecnología de los Alimentos (ICTA), Córdoba, Argentina; 2 Consejo Nacional de Investigaciones Científicas y Técnicas (CONICET), Instituto de Investigaciones Biológicas y Tecnológicas (IIByT, CONICET-UNC), Córdoba, Argentina

**Keywords:** Animal behaviour, Scale invariance, Power law, Time series

## Abstract

The behavioral dynamics within a social group not only could depend on individual traits and social-experience of each member, but more importantly, emerges from inter-individual interactions over time. Herein, we first present a dataset, as well as the corresponding original video recordings, of the results of 4 behavioral tests associated with fear and aggressive response performed on 106 Japanese quail. In a second stage, birds were housed with conspecifics that performed similarly in the behavioral tests in groups of 2 females and 1 male. By continuously monitoring each bird in these small social groups, we obtained time series of social and reproductive behavior, and high-resolution locomotor time series. This approach provides the opportunity to perform precise quantification of the temporal dynamics of behavior at an individual level within different social scenarios including when an individual showing continued aggressive behaviors is present. These unique datasets and videos are publicly available in Figshare and can be used in further analysis, or for comparison with existing or future data sets or mathematical models across different taxa.

## Abstract (summary)

**Table:** 

Measurement(s)	locomotor behavior trait • social interaction measurement
Technology Type(s)	video camera
Factor Type(s)	sex • day of testing
Sample Characteristic - Organism	Coturnix japonica
Sample Characteristic - Environment	laboratory environment

Machine-accessible metadata file describing the reported data: 10.6084/m9.figshare.10289750

## Background & Summary

The behavioral dynamics within a social group depends on many factors (i.e. individual traits, prior social-experience of its members, environmental context) and emerges from the interactions between its members over time. For example, individuals considered to be more aggressive toward conspecifics, may show more dominant behavior, and be more explorative, bold and active^1,2^. In farm animals it is well known that animals can be selected based on certain behavioral traits and this impacts, at least on a population level, on the overall behavioral performance in a wide variety of contexts. For example, quail selected by their high andrenocortical response to restraint, are more fearful in a wide variety of tests^3^ but also more aggressive in social groups, in comparison with those with low responsiveness^4^. Also, quail selected as chicks as highly sociable, are less fearful and less aggressive in social groups as juveniles than less sociable birds. Thus, the use of selection tests can favor a higher proportion of individuals with a desired characteristic^5^.

Although individual traits such as fearfulness and aggressiveness can affect the outcome of social interactions and the establishment of dominance hierarchies^6–8^, other factors such as previous fighting experience^9^, group size^10–12^ and housing conditions (i.e. size of box or cage^10,13^, presence or not of enrichment^14,15^) as well as the dynamical interaction between them^16^ can determine the social dynamics of a group. For instance, in large groups it has been proposed that tolerant social dynamics, that does not require individual recognition per se, emerges as the predominate social strategy^10,11^, while hierarchy formation is predominant in small groups^12^. Housing that is relatively small in relation to group size, can lead to high stocking density hence an increase of frequency of agonistic acts^10,13^.

In poultry, like other birds, within small groups hierarchies are established through a peck-order, according to which the animal that rank highest pecks at conspecifics and it is not pecked in return, and the opposite happens to the animals at the extreme bottom of the rank^17,18^. Hence, when two or more unacquainted adult birds are brought together, fights and pecks usually occur until each bird has established a dominance-subordination relationship with each other^12^. Thus, dominance is an emergent property that springs from the interaction of at least two individuals^19^, where a more aggressive bird in a specific environmental/social context becomes dominant. In this context, the study of hierarchical social groups in farm animals, and in poultry in particular^12,20,21^, has been widely addressed due to welfare implication. These welfare concerns arise from the observation in farms that, especially in small social groups, aggressive interactions target subordinates leading to high levels of social stress and in the worst cases to death.

Although important, the in-depth study on the behavioral patterns of individuals in their social environment is difficult from a methodological standpoint. In particular, tracking animals automatically within social groups present a unique difficulty, provided that animals in groups touch each other, move in paths that cross, and interact in complex ways, leading to an undesired switch of identities of unmarked individuals^22^. This is especially a problem when tracking poultry in groups, given that not only do animals frequently lay close to each other, but also during reproductive behavior male’s mount females (thus are literally on top of females) rendering automatic individualization during mounts impossible. Recent development of software has assessed this problem^22–24^. In particular, idTracker^22^ uses a multitracking algorithm that extracts a characteristic fingerprint from each animal in a video recording of a group. It then uses these fingerprints to identify every individual throughout the video. Tracking by identification minimizes propagation of errors, and thus correct identities can be maintained.

Once high-resolution individual tracking is achieved using specialized software, we are able to asses’ temporal patterns of locomotion of all individuals in the social group. Locomotor temporal patterns are particularly interesting given that they reflect both motivations to move (i.e. to feed, drink, or escape) and to remain immobile (i.e. when resting, fearful, threatened or are hiding). Moreover it is well known that locomotion does not occur randomly over time but rather presents long-term correlations (i.e. present behavior depends on past behavior^25^) and fractal dynamics (i.e. fluctuations occur on a broad range of time scales). These properties can be evaluated using the appropriate mathematical tools, thus providing insight on the temporal fractal complexity^26–28^. The degree of fractal complexity of behavior has been associated with health status^29–32^, stress^33–35^, welfare^26,36^, and environmental complexity^28,32^. Specifically, social stressors have been shown to induce changes in behavioral complexity^26,32,36^ highlighting the usefulness of this strategy in the study of organization of behavior within social groups.

Herein, in a first stage of the experimental setup we evaluate 106 Japanese quail (53 males and 53 females) in 4 experimental situations that can be associated with level of fearfulness or aggressiveness. In this context, longer latency to ambulate in a novel environment, longer tonic immobility reactions and more pronounced silence and inactivity during mechanical restraint have all been equated with increased fearfulness in several genetic lines of chickens and Japanese quail^6,37–43^. Aggressive behavior displayed during social interactions with an unknown conspecific or with a cagemate have been associated with levels of male aggressiveness^44–46^. In a second stage, social groups were arranged based on performance in preselection tests. The social groups were triads of 2 females and 1 male. This proportion 2:1 (female:male) allowed assessment of female—female as well as female-male interactions, while avoiding well documented violent male-male aggressions. Moreover, triads were used given that hierarchy can easily be visualized and it is well documented that in triads, predominately, linear hierarchy (i.e. if bird A dominates B and B dominates C, then A also dominates C) are established^16,47,48^. By continuously monitoring each bird in these social groups, we obtained time series of social and reproductive behavior and high-resolution locomotor time series. This approach provides the opportunity to perform precise quantification of the temporal dynamics of behavior at an individual level within their social environment including when one of the group members is showing continued aggressive behaviors. Elsewhere^49^ we show that subordinate animals (i.e., none or low levels of aggressive interactions; neutral groups) that are continuously pecked at during a 1 h period show quantitatively distinct dynamics of locomotion (i.e. lower level of fluctuation between immobility and mobility events, thus longer durations of events) in comparison to those that receive few or no aggression from conspecifics, deemed dominants. Moreover we show that subordinates also showed a high level of synchronization in locomotor pattern with the dominant member, likely reflecting a lack of “freedom” to perform locomotor behavior^49^. The data sets of all behavioral tests, the behavioral time series obtained in social groups of divergent characteristics described herein, as well as the corresponding original video recordings are publicly available on Figshare^50–53^. This data can be used to for comparison with existing or future data sets, and mathematical models developed in other species.

## Methods

In this section we describe in more detail the methods described in Alcala *et al*.^49^. All the procedures were in compliance with the Guide for the Care and Use of Laboratory Animals issued by the National Institute of Health (NIH Publications, Eighth Edition). Experimental protocol was approved by the Institutional Council for the Care of Laboratory Animals (CICUAL, Comité Institutional de Cuidado de Animales de Laboratorio) of the Instituto de Investigaciones Biologicas y Technologicas (IIByT, CONICET - Universidad Nacional de Córdoba).

### Animals and husbandry

The study was performed with Japanese quail (*Coturnix japonica*) a species widely used for studies covering neuroendocrine and social behaviors studies^54,55^. Also, they are considered an excellent laboratory model for the extrapolation of data to other poultry species with higher commercial relevance because of its high physiological similarity^39,54^. The animals were bred according to standard laboratory protocols^56,57^. Mixed-sex Japanese quail hatchlings were randomly housed in groups of 50–60 in white wooden brood boxes measuring 90 × 80 × 60 cm (length × width × height respectively) with a feeder along one wall, and 16 automatic nipple drinkers. A wire-mesh floor (1 cm grid) was raised 5 cm to allow the passage of excreta to the collection tray to facilitate cleaning and comfort of the animals, and a lid prevented the birds from escaping. Brooding temperature was 37.0 °C during the first week of life, with a weekly decline of 3.0 °C until room temperature (24 to 27 °C) was achieved. Food and water were provided *ad libitum*. The first week of life all animals were raised under the same standard conditions. Quail were subjected to a daily cycle of 14 h light (300 to 320 lx): 10 h dark (long photoperiod; photostimulated) throughout the study, with the exception of Photocastrated stimulus birds (for the Social interaction test, see below) that were submitted to a short photoperiod light cycle (06 h light: 18 h dark) beginning at 4 weeks of age until testing ended^58^.

At 28 days of age, test animals were sexed by plumage coloration, marked with a numbered wing band and randomly housed in pairs of 1 male and 1 female in cages of 20 × 40 × 20 cm (width × length × height respectively).

If an animal showed any indication of illness or escaped from their cage during rearing, they and their companion cagemate were completed excluded from the experiment.

### Preselection of quail

**Fig. 1:**
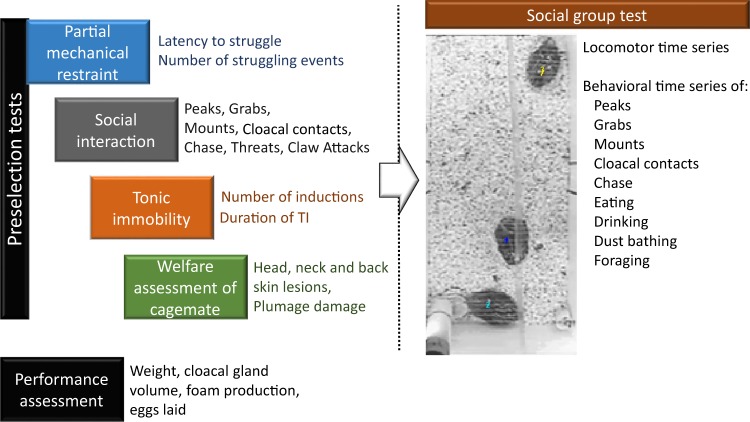
Schematically representation on experimental design. On the left the 4 preselection tests assed, namely, Partial mechanical restraint, Tonic Immobility, Social Interaction and Welfare assessment, and performance assessment. On the right a picture of the social group tests, where the individuals (1 male and 2 females) are observed as well as the feeder in the bottom left corner and automatic nipple in bottom right corner of the apparatus. The variables registered in each test is written in the proximity of the test name.

One-hundred six quail were first evaluated in 4 preselection tests, separated between each other roughly by 30 days in order to favor independence between tests. These tests were used as a preselection criterion for social group testing. All data registered during the preselection tests are available in file “PreselectionTestsQuail.xls” and stored in the public repository Figshare^52^. Original video recordings are also available^53^. A schematically representation of the experimental design, tests and variables registered is shown in Fig. 1. In each test the order of testing of cages was randomized avoiding evaluation of adjacent cages consecutively. Both birds of the cage were always evaluated simultaneously. Moreover, the experimenters were always blinded regarding the prior history of the animals allocated in each group. With the exception of the tonic immobility test, all tests were recorded onto a computer and video recordings were analyzed the following days after testing by one previously trained experimenter.

#### Partial mechanical restraint

**Fig. 2:**
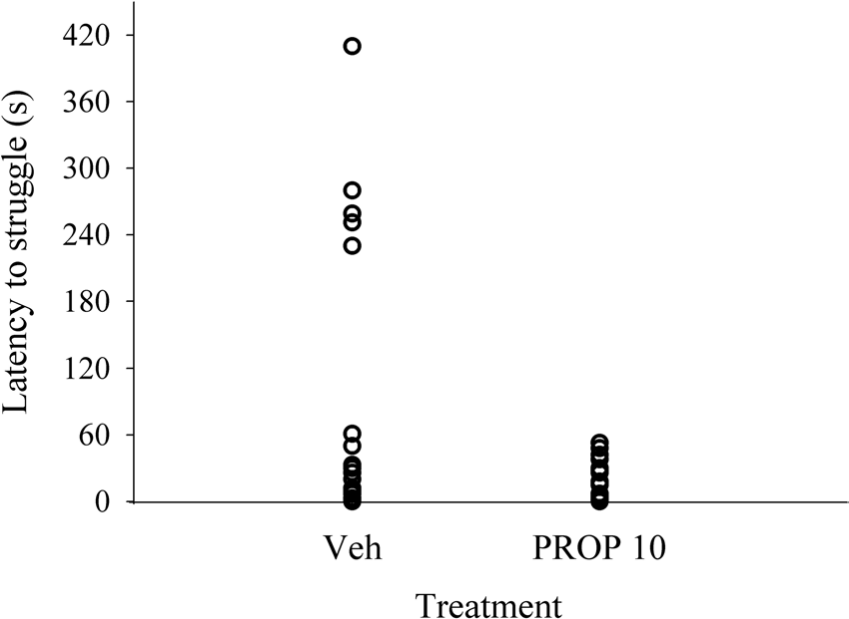
Pilot study showing 60 s threshold for latency to stuggle during Partial Mechanical Restraint in Japanese quail treated with a subsedative anxiolytic dose of propofol. Latency to struggle during partial mechanical restraint in juvenile (31–32 days old) Japanese quail treated with either vehicle (i.e. distilled water and 20% Tween-80 (Sigma Chem. Co.)) or a propofol 10 mg/kg^57^. Birds were placed into the restraint apparatus ten min after intraperitoneal administration. Note that birds treated with Vehicle showed struggling latencies from 0–300 s while none of the birds treated with propofol showed latencies above 60 s. Dotted line indicates this 60 s threshold.

This test has been proposed as a method to measure fear in quail^6,59,60^. Moreover, in juveniles, subsedative^57^ anxiolytic doses of Propofol have shown to reduce struggling (see below) to durations bellow 60 s (See pilot study results in Fig. 2). This test was performed at 40 days of age. This test consists in restricting the movement of the animal between two walls of a melamine box of 20 × 10 cm (height per width, respectively) with the characteristic that the front wall was made of glass (it allows the visualization of the animal and video recording of its behavior), and the back wall was adjustable to induce immobility in such a way that the animal cannot open the wings, but can move the head and legs^9^. The experimenters retreated out of the birds’ sight, and the test was during 5 minutes recorded with a video camera place in front of the box. All the birds were tested in 31 batches of 4 animals each, where the birds had no visual or physical contact between each other. The video was analyzed manually, and the following variables were recorded: the **latency to struggle** considered as time between the initiation of restraint until the first struggling episode (defined as the birds making fast movement with their legs when aiming to escape from the test apparatus) and the **number of struggles** events during the observation period. The struggle events were considered different if they were separated by 5 s or more. The immobility of the animal during the test has been widely considered in the literature as an indicator of fear^6,59,60^. Struggling during such restraint is known to be more pronounced in genetic lines of quail showing low rather than high levels of underlying fearfulness^6,40^. Those whose latency of struggle was >60 s were considered as fearful (Fig. 2)^61^.

#### Social interaction (SI) test

This test is described in detail in Caliva *et al*. (2017) and measures levels of aggressiveness towards a non-aggressive photocastrated male opponent in a novel environment^58^. The SI test was performed over a five day period, between 70 and 74 days of age. Briefly, the SI test consists in a 5 min encounter between an unfamiliar test adult bird and a photocastrated stimulus adult male, in the presence of the test bird’s cagemate (audience). A video-camera was positioned 1 m above the apparatus and connected to a computer that allowed constant monitoring and recording during the test while out of the sight of the birds. Using commercially available behavioral tracking software (ANY-maze™, 2015) the number of events (i.e. continuous time performing the behavior separated from the following event by at least 5 s), duration of behavior (i.e. seconds performing behavior) and latency to initiate behavior (i.e. time from the start of the test until bird shows the first event) of the following aggressive behaviors were recorded:

**Pecks**: when one bird raises its head and vigorously pecks the other bird’s body (usually on the head).

**Grabs**: when a male catches (“grabs”) with their beak the neck or head region of the female.

**Mounts**: while performing a grab, the male approaches a female from behind, and places both feet on the dorsal surface of its torso, stepping over the females’ tail (adapted from^62^).

**Cloacal contacts**: during mounting, the male lifts his tail and tilts his pelvis underneath the other bird and briefly presses its cloaca against the female (adapted from^62^).

**Threats**: one bird stands with its neck and head raised in front of the other bird that usually has its’ head at a lower level than the first (adapted from^62^).

**Chase**: a bird runs after another that is escaping (adapted from^63^).

Herein, when grabs, mounts or cloacal contacts were performed by one male towards another male, they were considered as aggressive behaviors^64^. Birds that performed more than 5 aggressiveness behaviors were considered aggressive, and birds that did not perform any aggressive behavior towards the photocastrated opponent were considered non-aggressive^58^. If during the interaction a quail received more than 5 consecutive aggressive pecks, showed a clear and continued escaping (retrieval) behavior, and/or showed any sign of physical damage, the interaction was immediately interrupted^65^. Caliva *et al*. (2017) showed that in the SI test only 8% of the photostimulated females showed clear signs of aggression towards photocastrated opponent. The authors proposed that this is most likely due to the short duration of the test (up to 5 min), and that longer test durations are needed in females to observe significant aggressiveness (i.e. 3 h tests are performed in hens to establish dominance^16,48,66^). Thus, expression of aggressive behavior in females was very low.

#### Tonic immobility (TI)

The TI test was performed at 100 days of age. According to Jones^67^ this test induces an unlearned antipredator response that is triggered by a brief period of physical restraint. In the test the individual was place in the left lateral decubitus and hold for 15 seconds (the necessary time required to unleash the muscular immobilized tonic behavior), holding him with both hands against a support base (one hand on the head and another in the body). We recorded the **number of inductions** to achieve an immobility of at least 10 seconds and the **duration of TI** once induced. Maximum duration of TI was fixed at 5 minutes. Duration of TI implies both a behavioral and physiological response modulated by frightening situations and is considered as a measure of the level of fearfulness^68,69^. Thus, a long duration of TI and a smaller number of necessary inductions is indicative of a high level of fear as opposed to a short response^67^. If IT was not attained after 5 successive attempts, the bird was considered not to be susceptible and scores of 0 were given for TI duration. Thus “non-fearful” birds were selected based on those that needed 4 or more inductions, while the birds considered “fearful” required a single induction in the test.

#### Welfare assessment

At 96 and 108 days of age female skin lesions and plumage status were evaluated following a procedure proposed by Pellegrini *et al*.^46^ that is an adapted version of the protocol proposed by Welfare Quality® consortium^70^. Pellegrini *et al*. showed in Japanese quail that male aggressions toward a female cagemate can predict aggressiveness toward unknown conspecifics^46^. **Head**, **neck and back skin lesions** were determined using a score scale from 0 to 2 where “0” represents no lesions (punctiform damage <0.25 cm diameter) or scratches, “1” represents less than 3 lesion or scratches, and “2” reflects 3 or more lesion or scratches. **Head**, **neck and back plumage damage** was also determined using a score scale from 0 to 2 as follows: “0” represents individuals with no plumage damage or slight wear (only single feathers lacking), “1” represent individuals with one or more body parts that have moderate wear (i.e. damaged feathers worn or deformed) or one or more featherless areas <1.5 cm in diameter at the larger extent and “2” corresponded to individuals that have at least one featherless area >1.5 cm in diameter at the largest extent. None of the males showed plumage damage or lesions, thus, only male aggression towards females were considered in this analysis. Plumage damage induced by males (score > 0) to their female cagemates were considered as indicative of male aggressiveness^46^. Non-aggressive males were those in which no plumage damage was seen in female cagemates^46^. It should be noted that if at any point in the study a bird showed severe lesions they were separated from their cagemates and thus both cagemates were eliminated from the study in order to protect the welfare of the animals. Due to this systematic standard laboratory procedure, very aggressive birds were excluded from the study even at a young age.

### Performance assessment

**Fig. 3:**
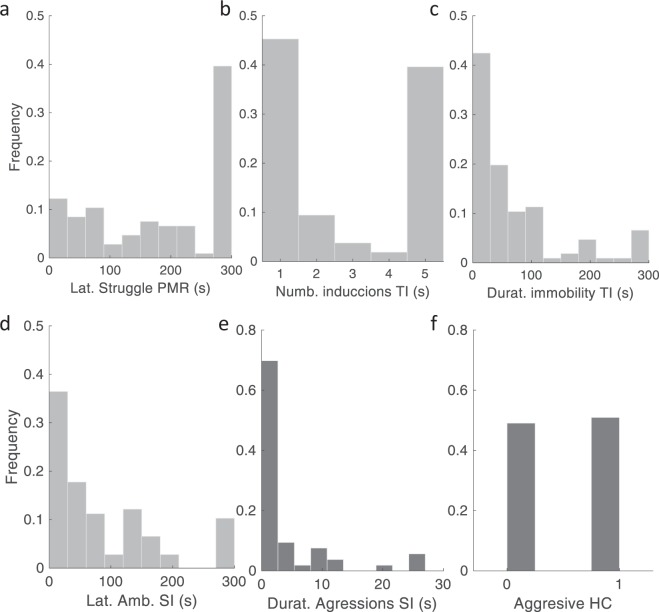
Frequency distribution of preselection test variables. Frequency distribution of (**a**) latency to struggle during Partial Mechanical Restraint, (**b**) number of inductions in Tonic Immobility test and (**c**) the duration of the tonic immobility, (**d**) latency to ambulate during the first stage and (**e**) total duration of aggressions in the Social Interaction test, and (**f**) aggressive in home cage valued by welfare assessment of cagemate. Frequency distribution of variables “a”-“d” are shown for data pooled from females and males, while variables “e”-“f” are only from males. A total of 106 animals were studied, half female and half males.

Birds were weighed at 28 days of age. The weight of birds transferred to cages ranged between 100–150 g. Thereafter, weight and male cloacal gland width and length, and male foam production were recorded weekly until 9 weeks of age, when all males showed completed gonadal development (Cloacal gonadal volume CGV > 1000 mm^3^). Cloacal gland volume was estimated as (4/3 × 3.5414 × a × b2), where a = 0.5 × length, and b = 0.5 × width^71^. Foam production was quantified by subjective scaling of the amount of foam ejected upon manual expression (squeezing) of the foam gland, using a scale of 1 (no foam expressed) to 5 (maximum amount of foam expressed). Female quail egg production was monitored throughout the study and all females reached peak egg production. All birds were also weighed after the last behavioral test at 92 days of age, and male cloacal gland size and foam production also assessed. This data is available in file “PreselectionTestsQuail.xls” and stored in the public repository Figshare^52^, and Frequency distribution of variables are shown in Fig. 3.

### Principal component analysis of preselection tests

**Fig. 4:**
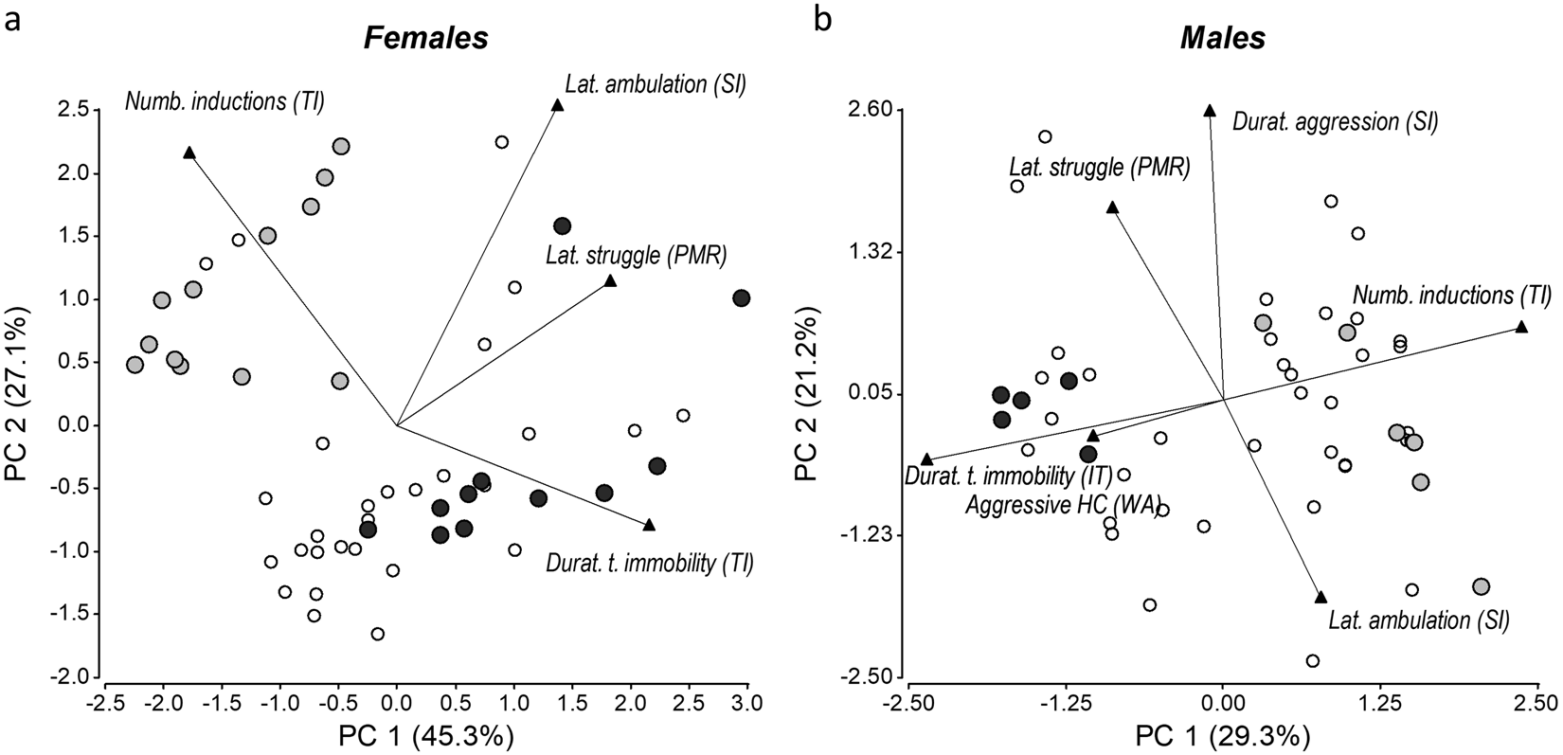
Principal components analysis biplot of the preselection tests data. Each point represents (**a**) a female or (**b**) a male quail. Variables are ploted as vectors from the origen ending in black triangles. Birds used for social groups type A are shown in black circles while those used for type B are shown in gray circles (see main tet for definition). The percent of the eigenvalues of each PC are shown in brackets next to each component. Only variables with low levels (R^2^ < 0.40) of correlations between them were used in the analysis: latency to struggle during Partial Mechanical Restraint (*Lat*. *struggle (PMR)*), number of inductions (*Numb*. *Inductions (TI)*) and the duration of the tonic immobility (*Durat*. *t*. *immobility (TI)*) during the Tonic Immobility test, latency to ambulate during the first stage (*Lat*. *ambulation (SI)*) and total duration of aggressions (*Durat*. *aggression (SI)*) in the Social Interaction test, and aggressive in home cage valued by welfare assessment of cagemate (*Aggressive HC (WA)*). A total of 106 animals were studied, half female and half males.

Principal Component Analysis biplots of the preselection tests are shown in Fig. 4, and illustrates the relationship between variables, and selection criteria. Statistical independence (R2 = 0.04 and ~90° angles in the PCA biplot (lines in Fig. 4)) was observed between variables of two different tests, namely latency to struggle during partial mechanical restraint and number of inductions for tonic immobility. However, 74% of females that were fearful in the tonic immobility test (i.e. only needed one induction) also showed low level of struggling (i.e. ≤3 struggling bouts), in comparison to 34% (P < 0.05, 2-tailed proportion test) that showed low struggling and were found to be less fearful (i.e. tonic immobility was not induced or only after 5 inductions). This was not evident in males (65% and 68% fearful and non-fearful during tonic immobility, respectively). In all, these results show that, at least in females, highly fearful birds during tonic immobility on average were also more fearful during restraint.

Considering results obtained in preselection test birds were classified in 2 types: 1) Type A (Fig. 4 black circles) had birds that were fearful in both the Tonic immobility and the partial mechanical restraint test. These males also were aggressive in the Social interaction test or their female cagemate showed higher scores during Welfare assessment. 2) Type B (Fig. 4 gray circles) had females and males that were not fearful in the Tonic immobility test, and males that tested non-aggressive in both the Social interaction test and in their home cage. No differences between body weight, cloacal gland volume or foam production was observed between types. It should be noted that there are males that were not used for the social group tests (Fig. 4 open circles) because they were used in a separate neurobiology experiment.

### Social group testing

Novel social groups (2 females: 1 male) of animals (156–171 days old) that behaved coherently in the 4 preselection tests described in the previous section, thus half of the 12 social groups evaluated had type A birds and the other half type B birds (see previous subsection). Social groups were housed in a white wooden apparatus measuring 80 × 40 × 40 cm (width × length × height, respectively) with wood-shavings on the floor. A feeder and an automatic nipple drinker were positioned in opposite corners of the apparatus (Fig. 1, left and right bottom corner of box in the photograph, respectively). Nylon monofilament line was extended over the top of the boxes with a 1 cm separation in order to prevent the birds from escaping without interfering with their visualization. A video camera was suspended 1.5 m above the box. Since only 4 social groups could be tested simultaneously, the setup was repeated 3 consecutive times. For convenience, boxes in which each social group were placed were numbered from 1 to 12. Boxes 1–4 were tested simultaneously first, 5–8 second and 9–12 last. Video recordings are publicly available on Figshare^53^.

We used IdTracker^22^ in MATLAB R2017a to register x, y coordinates of each animal within the social group during a 1 h period immediately after being placed in the test apparatus between 9 and 10 am, and 48 hours after testing began. Locomotion was than estimated at 0.5 s intervals (7200 time intervals) using customized code Locomotion.m that calculates the distance moved by the animal, converts distance expressed in pixel to centimeters, and if the distance moved is above a threshold of 1 cm that the animal is considered mobile^72^. Thus, the recorded behavioral data is expressed in the form of a time series of mutually exclusive states. At any given time, if the bird was moving a number one was recorded or a zero if immobile. These locomotor time series are publicly available and stored in the public repository Figshare^51^.

Time series of non-locomotor behaviors were obtained through visual observation of video recordings using as an interface ANY-MAZE@ to register behavior. For each bird, when the specific behavior was performed the corresponding key was pressed until the bird finished performing the behavior, thus a binary time series, x_i,_ sampled at up to 2 data points per second was constructed for each behavior.

$${x}_{i}\left\{\begin{array}{c}0\left(not\,performing\,the\,behavior\right)\\ 1\left(performing\,behavior\right)\end{array}\right.$$

Only one observer recorded all data in order to avoid inter-individual variability. Prior to video analysis observer performed training sessions than consisted in analyzing the same three behavioral video at least two times. Then, reliability was estimated to be >95%. (formula: number of agreements/number of agreements + number of disagreements).

The following behaviors were recorded: *Pecks*, *Grabs*, *Mounts*, *Cloacal contacts*, *Threats*, *Chase*, as described previously for the SI test, and additionally, *Foraging*: pecking at the ground or actively moving litter with beak, *Feeding*: peaking at food in the feeding trough, and *Dust bathing*: vertical wing shakes in a lying position^73^. From the behavioral time series both frequency and durations of behaviors can be easily estimated. These behavioral time series are also publicly available and stored in the public repository Figshare^50^.

## Data Records

Original video recordings^53^ of Partial Mechanical Restraint, Social Interaction test and Social groups are provided in avi or mod format. File names include the abbreviation, PMR, SI or Box, respectively. For video file of the Partial Mechanical Restraint and Social Interaction tests file names also include the ID of the animals tested. In the case of social groups box number (1–12) and the day of testing (day1 or day3) is provided in the file name.

The results of the preselection tests are presented in the excel file “PreselectionTestsQuail.xls” stored in the public repository Figshare^52^ with the headers of the columns representing the variable analyzed in each test for each animal (rows). Table 1 presents all the column headers as well as a brief definition of the variable.

**Table 1:** Column headers and definition of morphometric and behavioral variables recorded during preselection tests publicly available on Figshare^52^ in excel file “PreselectionTestQuail.xls”.

Variable		Definition
Perform. assessment	Body weight (g)	Animal weight using balance Ohaus Scout- Pro®(SP601).
	CGV (mm^3^)	Cloacal gland volume estimated as (4/3 × 3.5414 × *a* × *b*)^71^, where *a* = 0.5 × length, and *b* = 0.5 × width of cloacal gland
	Foam product.	Subjective scaling of the amount of foam ejected upon manual expression of the foam gland, using a scale of 1 (no foam expressed) to 5 (maximum).
Partial mechanical restraint	Lat. struggle (s)	Time in seconds between the initiation of restraint until the first struggling episode. If struggling was not observed, 300 was recorded.
	N. of struggles	Number of struggles during the observation period.
Social interaction test	N. of pecks	Number of events when one bird raises its head and vigorously pecks the other bird’s body.
	Dur. of pecks (s)	Seconds spent pecking at opponent.
	N. of grabs	Number of events when a bird catches (“grabs”) with their beak the neck or head region of the other bird.
	Dur. of grabs (s)	Seconds spent performing grabs towards opponent.
	N. of mounts	Number of events while performing a grab, the bird approaches the other bird from behind, and places both feet on the dorsal surface of its torso, stepping over the other birds’ tail.
	Dur. of mounts (s)	Seconds spent performing mounts towards opponent.
	N. of C.C.	Number of events during mounting, the bird lifts his tail and tilts his pelvis underneath the other bird and briefly presses its cloaca against the other bird.
	Dur. of C. C. (s)	Seconds spent performing cloacal contacts.
	N. of threats	Number of events when one of the birds raises its head and neck rapidly, moves forward and backward vigorously in the direction of the opponent without making physical contact.
	Dur. of threats (s)	Seconds spent performing threats towards opponent.
	N. of chases	Number of events a bird runs after another that is escaping.
	Dur. of chases (s)	Seconds spent chasing opponent.
Tonic immob Test	N. of inductions	Number of inductions to achieve an immobility of at least 10 s.
	Dur. of TI (s)	Duration of TI once induced. Maximum duration fixed at 5 min.
Welfare assessment at 96 days of age and Welfare assessment at 108 days of age	Lesions: head, neck and back skin	Score from 0 to 2: 0 = no lesions (punctiform damage <0.25 cm diameter) or scratches; 1 = three lesion or scratches; 2 = three or more lesion or scratches on head, neck or back. Measured at 96 or 108 days of age, respectively.
	Plumage damage: head, neck and back skin	Score scale from 0 to 2 as follows: 0 = no plumage damage or slight wear (only single feathers lacking) in head neck and back; 1 = moderate wear (i.e. damaged feathers worn or deformed) or one or more featherless areas <1.5 cm in diameter at the larger extent; 2 = at least one featherless area >1.5 cm in diameter at the largest extent. Measured at 96 or 108 days of age.
	Welfare of cagemate	Binary score, 0 = absence of injuries in cage mate, 1 = presence of at least one injury greater than 1 cm in diameter in cagemate

All time series from this study are stored in Figshare as text files (.txt). For practical purposes, locomotor data obtained from IdTracker are in separate files^51^ from the behavioral data time series obtained from AnyMaze^52^. Locomotor data consists of a single column of data sampled at 0.5 s intervals (as explained previously). For the behavioral data the first column refers to the time while the following columns refer to the behavioral data. Considering that the 36 animals were evaluated in 12 mixed-sex groups of 3 birds in individual experimental boxes on the first hour (Day1) and 48 hours (Day3) after test initiation, each subject quail was identified by their experimental group number (Box), ID number of wing band, and sex (femaleA, femaleB or male). In the case of females, an indication A or B is used to discriminate between the two. In the file name, an indication of the corresponding bird is also provided as “BoxN°_IDN°_sex_DayN°” (Table 2) for recorded obtained.

**Table 2:** Overview of the data files uploaded to Figshare^50,51^ grouped in file sets according to time series type (locomotor, and behavioral) recorded from the 2 females and 1 male quail studied during a one-hour period in each of the 12 social groups housed in boxes after 48 hours of habituation to the social environment (Day3).

Box	ID	Sex	Locomotor Time Series	Behavioral Data Time Series
1	6289	Female A	Box 1_ID6289_femaleA_Day3.txt	BEH_Box 1_ID6289_femaleA_Day3.txt
1	1642	Female B	Box 1_ID1642_femaleB_Day3.txt	BEH_Box 1_ID1642_femaleB_Day3.txt
1	6287	Male	Box 1_ID6287_male_Day3.txt	BEH_Box 1_ID6287_male_Day3.txt
2	1677	Female A	Box 2_ID1677_femaleA_Day3.txt	BEH_Box 2_ID1677_femaleA_Day3.txt
2	6258	Female B	Box 2_ID6258_femaleB_Day3.txt	BEH_Box 2_ID6258_femaleB_Day3.txt
2	1172	Male	Box 2_ID1172_male_Day3.txt	BEH_Box 2_ID1172_male_Day3.txt
3	4290	Female A	Box 3_ID4290_femaleA_Day3.txt	BEH_Box 3_ID4290_femaleA_Day3.txt
3	6280	Female B	Box 3_ID6280_femaleB_Day3.txt	BEH_Box 3_ID6280_femaleB_Day3.txt
3	4254	Male	Box 3_ID4254_male_Day3.txt	BEH_Box 3_ID4254_male_Day3.txt
4	4295	Female A	Box 4_ID4295_femaleA_Day3.txt	BEH_Box 4_ID4295_femaleA_Day3.txt
4	4238	Female B	Box 4_ID4238_femaleB_Day3.txt	BEH_Box 4_ID4238_femaleB_Day3.txt
4	4299	Male	Box 4_ID4299_male_Day3.txt	BEH_Box 4_ID4299_male_Day3.txt
5	1178	Female A	Box 5_ID1178_femaleA_Day3.txt	BEH_Box 5_ID1178_femaleA_Day3.txt
5	1196	Female B	Box 5_ID1196_femaleB_Day3.txt	BEH_Box 5_ID1196_femaleB_Day3.txt
5	1684	Male	Box 5_ID1684_male_Day3.txt	BEH_Box 5_ID1684_male_Day3.txt
6	4232	Female A	Box 6_ID4232_femaleA_Day3.txt	BEH_Box 6_ID4232_femaleA_Day3.txt
6	6268	Female B	Box 6_ID6268_femaleB_Day3.txt	BEH_Box 6_ID6268_femaleB_Day3.txt
6	6278	Male	Box 6_ID6278_male_Day3.txt	BEH_Box 6_ID6278_male_Day3.txt
7	4300	Female A	Box 7_ID4300_femaleA_Day3.txt	BEH_Box 7_ID4300_femaleA_Day3.txt
7	4237	Female B	Box 7_ID4237_femaleB_Day3.txt	BEH_Box 7_ID4237_femaleB_Day3.txt
7	4281	Male	Box 7_ID4281_male_Day3.txt	BEH_Box 7_ID4281_male_Day3.txt
8	4271	Female A	Box 8_ID4271_femaleA_Day3.txt	BEH_Box 8_ID4271_femaleA_Day3.txt
8	4230	Female B	Box 8_ID4230_femaleB_Day3.txt	BEH_Box 8_ID4230_femaleB_Day3.txt
8	4249	Male	Box 8_ID4249_male_Day3.txt	BEH_Box 8_ID4249_male_Day3.txt
9	6283	Female A	Box 9_ID6283_femaleA_Day3.txt	BEH_Box 9_ID6283_femaleA_Day3.txt
9	1643	Female B	Box 9_ID1643_femaleB_Day3.txt	BEH_Box 9_ID1643_femaleB_Day3.txt
9	6252	Male	Box 9_ID6252_male_Day3.txt	BEH_Box 9_ID6252_male_Day3.txt
10	1181	Female A	Box 10_ID1181_femaleA_Day3.txt	BEH_Box 10_ID1181_femaleA_Day3.txt
10	1195	Female B	Box 10_ID1195_femaleB_Day3.txt	BEH_Box 10_ID1195_femaleB_Day3.txt
10	6288	Male	Box 10_ID6288_male_Day3.txt	BEH_Box 10_ID6288_male_Day3.txt
11	4264	Female A	Box 11_ID4264_femaleA_Day3.txt	BEH_Box 11_ID4264_femaleA_Day3.txt
11	4500	Female B	Box 11_ID4500_femaleB_Day3.txt	BEH_Box 11_ID4500_femaleB_Day3.txt
11	4273	Male	Box 11_ID4273_male_Day3.txt	BEH_Box 11_ID4273_male_Day3.txt
12	4280	Female A	Box 12_ID4280_femaleA_Day3.txt	BEH_Box 12_ID4280_femaleA_Day3.txt
12	4278	Female B	Box 12_ID4278_femaleB_Day3.txt	BEH_Box 12_ID4278_femaleB_Day3.txt
12	4499	Male	Box 12_ID4499_male_Day3.txt	BEH_Box 12_ID4499_male_Day3.txt

## Technical Validation

All data analysis and technical validation was performed by one observer both in Any-Maze as well as in IdTracker. In both cases the observer was blinded regarding the prior history of the animals allocated in each group. One of the advantages of IdTracker is that the researcher can perform visual observation of the tracking performed on each frame analyzed using the complementary software IdPlayer. A number (see Fig. 1) in the center of the of the animal a number indicates the identity of the bird. In the case of identification errors, they were corrected manually using this software. In order to validate the correct tracking and identification of the animal, visual observations of tracking were performed for all birds. The high contrast between the white, well illuminated, box and the dark brown quail feathers facilitated an accurate tracking of the animal. Also animals had small white markings of their backs that allowed identification from video recordings.

Behavioral data sets were collected using the commercially available ANY-maze™ Video Tracking System software that can be downloaded at www.anymaze.com. Since in this software keystrokes allow observer to register manually behaviors from video recording, a validation period to guarantee reproducibility was first performed. Observer performed validation sessions than consisted in analyzing the same three behavioral video at least two times. Then, reliability was estimated to be >95%. (formula: number of agreements/number of agreements + number of disagreements).

## Data Availability

IdTracker^22^ is a videotracking software that keeps the correct identity of each individual during video behavioral analysis and is publicly available at http://www.idtracker.es/. ANY-MAZE@ is a licensed video tracking program, that can be downloaded from http://www.anymaze.co.uk/. The customized Matlab code customized code Locomotion.m in publicly available on Figshare^72^.
